# The authors reply

**DOI:** 10.1590/0102-311XEN146623

**Published:** 2023-10-23

**Authors:** Inês Rugani Ribeiro de Castro, Luiz Antonio dos Anjos, Elisa Maria de Aquino Lacerda, Cristiano Siqueira Boccolini, Dayana Rodrigues Farias, Nadya Helena Alves-Santos, Paula Normando, Maiara Brusco de Freitas, Pedro Gomes Andrade, Neilane Bertoni, Raquel Machado Schincaglia, Talita Lelis Berti, Letícia B. Vertulli Carneiro, Gilberto Kac

**Affiliations:** 1 Instituto de Nutrição, Universidade do Estado do Rio de Janeiro, Rio de Janeiro, Brasil.; 2 Departamento de Nutrição Social, Universidade Federal Fluminense, Niterói, Brasil.; 3 Instituto de Nutrição Josué de Castro, Universidade Federal do Rio de Janeiro, Rio de Janeiro, Brasil.; 4 Instituto de Comunicação e Informação Científica e Tecnológica em Saúde, Fundação Oswaldo Cruz, Rio de Janeiro, Brasil.; 5 Instituto de Estudos em Saúde e Biológicas, Universidade Federal do Sul e Sudeste do Pará, Belém, Brasil.; 6 Divisão de Pesquisa Populacional, Instituto Nacional de Câncer José Alencar Gomes da Silva, Rio de Janeiro, Brasil; 7 Instituto de Estudos em Saúde Coletiva, Universidade Federal do Rio de Janeiro, Rio de Janeiro, Brasil.

We appreciate the commentators’ reflections on the study entitled *Nutrition Transition in Brazilian Children Under 5 Years Old from 2006 to 2019* ^1^ *.* Next, we will describe our reflections on three central points that recurrently and convergently emerged from this set of comments.

The first point we would like to highlight is the recognition of the improvement of living conditions and nutrition indicators and the reduction of regional, schooling, and maternal race/skin color inequalities from 2006 to 2019 ^2^ ^,^ ^3^ ^,^ ^4^ ^,^ ^5^ ^,^ ^6^. This recognition is followed by warnings that Brazil still had much to do to fully overcome these inequalities in 2019, an even more evident need due to the effects of the dismantling of public policies Brazil experienced from 2016 to 2022 together to those effects of the COVID-19 pandemic. In fact, the history of our country is marked by a project for a deeply unequal society that daily violates the population’s basic human rights of the majority of the population to perpetuate the economic and social privileges of an elite. Overcoming these inequalities depends not only on technically well-designed public policies based on robust scientific evidence and free of conflicts of interest but also on decision-makers’ political will, the organization and pressure of organized civil society toward a rights-guaranteeing agenda, and governance processes and structures that expand and consolidate democracy and social participation in the formulation, monitoring, and evaluation of State public policies (rather than government ones), as has historically happened.

Pérez-Escamilla ^3^ regrets that the *Brazilian National Survey on Child Nutrition* (ENANI-2019) could not analyze the temporal trends of the indicators it studied according to the time periods in which different political administrations governed the country. This limitation is not intrinsic to ENANI-2019, it stems from the absence of nationwide studies on food and child nutrition from 2006 to 2019. Still, complementary analyses of the ENANI-2019 results regarding stunting in this *Supplement* ^7^ and evidence on public policies produced in the last two decades in Brazil indicate how much the period from 2003 to 2015 (which refer to Lula’s and Rousseff’s governments) - marked by the expansion of public policies to guarantee rights and spaces for participation and social control - virtuously improved living conditions and reduced inequalities in Brazil and how much the dismantling of these public policies from 2016 to 2022 (Temer’s and Bolsonaro’s governments) harmed the Brazilian population, especially its more vulnerable segments ^8^ ^,^ ^9^ ^,^ ^10^. These results corroborate the assertion that overcoming inequalities depends on political will, the strength of organized civil society, and democratic practices in the process of conducting public policies.

Secondly, we would like to address the challenge of preventing and dealing with all forms of childhood malnutrition: hunger, malnutrition, micronutrient deficiencies, early weaning, and overweight. The commentators ^2^ ^,^ ^3^ ^,^ ^4^ ^,^ ^6^ offered some suggestions to deal with this challenge: synergistic and convergent intersectoral public policies, which combine structure measures with responses to immediate needs and which confront the predatory practices of large corporations, especially those linked to food systems assuming the Brazilian dietary guidelines ^11^ ^,^ ^12^ as guiding instruments and inducers of these public policies. We should also emphasize the centrality of policies that contribute to overcoming gender inequalities related to child care. Note that to overcome such inequalities will also require politicians’ political will, civil society’s organization and strength, and democratic processes to further this public policy agenda. The national conferences on health and food and nutrition security (and others that will take place up to December 2023) in Brazil configure important moments to guide demands, guidelines, and priorities that can influence public policy plans under such an intersectoral, convergent, and synergistic perspective.

The third point concerns the production of knowledge on child feeding and nutrition in Brazil. Commentators’ comments ^2^ ^,^ ^5^ praised the relevance of conducting national surveys to monitor trends and inequalities and the scientific rigor of ENANI-2019. They also highlighted the importance of complementing national surveys with studies aimed at specific populations, such as Indigenous peoples, quilombolas, and rural communities. In fact, despite the contribution of ENANI-2019 to knowledge about the food and nutrition profile of Brazilian children, the dynamism of eating habits and living conditions and the great inequalities marking Brazil point to the need for regular national surveys complemented by studies with more vulnerable groups.

The Brazilian National Food and Nutrition Security Policy (PNSAN) and the Food and Nutrition Security System (SISAN) provide for the production of evidence on food and nutrition ^13^ ^,^ ^14^. The Brazilian academic community can conduct these studies and has large experience in household-based population surveys. However, how regularly national studies are conducted depends (again) on the political will of the public power to ensure adequate financial resources. It is also necessary to articulate the planning of these studies toward complementarity to optimize financial resources and work processes. The upcoming 2024 edition of ENANI will be of paramount importance to monitor the evolution of Brazilian children’s food and nutrition profile. It will also provide information on public policies in this context of resumption of public policies based on an agenda to guarantee rights in Brazil.

Finally, we should say that the commented article we discussed brings a first set of evidence from ENANI-2019. The *Supplement* encompassing it offers a mosaic of results, including indicators on feeding for children 6-23 months of age, which Neves ^2^ claimed have found lacking. Additional results supporting the understanding of this complex phenomenon that is child feeding and nutrition in Brazil will be published in due course.
